# *APOBEC3G* Polymorphism as a Selective Barrier to Cross-Species Transmission and Emergence of Pathogenic SIV and AIDS in a Primate Host

**DOI:** 10.1371/journal.ppat.1003641

**Published:** 2013-10-03

**Authors:** Annabel Krupp, Kevin R. McCarthy, Marcel Ooms, Michael Letko, Jennifer S. Morgan, Viviana Simon, Welkin E. Johnson

**Affiliations:** 1 Institut für Klinische und Molekulare Virologie, Friedrich-Alexander-University Erlangen-Nuremberg, Erlangen and Nuremberg, Germany; 2 Department of Biology, Boston College, Chestnut Hill, Massachusetts, United States of America; 3 Harvard Program in Virology, Harvard Medical School, Boston, Massachusetts, United States of America; 4 Department of Microbiology, Icahn School of Medicine at Mount Sinai, New York, New York, United States of America; 5 Global Health and Emerging Pathogens Institute, Icahn School of Medicine at Mount Sinai, New York, New York, United States of America; 6 Division of Infectious Diseases, Department of Medicine, Icahn School of Medicine at Mount Sinai, New York, New York, United States of America; Fred Hutchinson Cancer Research Center, United States of America

## Abstract

Cellular restriction factors, which render cells intrinsically resistant to viruses, potentially impose genetic barriers to cross-species transmission and emergence of viral pathogens in nature. One such factor is APOBEC3G. To overcome APOBEC3G-mediated restriction, many lentiviruses encode Vif, a protein that targets APOBEC3G for degradation. As with many restriction factor genes, primate *APOBEC3G* displays strong signatures of positive selection. This is interpreted as evidence that the primate *APOBEC3G* locus reflects a long-term evolutionary “arms-race” between retroviruses and their primate hosts. Here, we provide direct evidence that APOBEC3G has functioned as a barrier to cross-species transmission, selecting for viral resistance during emergence of the AIDS-causing pathogen SIVmac in captive colonies of Asian macaques in the 1970s. Specifically, we found that rhesus macaques have multiple, functionally distinct APOBEC3G alleles, and that emergence of SIVmac and simian AIDS required adaptation of the virus to evade APOBEC3G-mediated restriction. Our evidence includes the first comparative analysis of APOBEC3G polymorphism and function in both a reservoir and recipient host species (sooty mangabeys and rhesus macaques, respectively), and identification of adaptations unique to Vif proteins of the SIVmac lineage that specifically antagonize rhesus APOBEC3G alleles. By demonstrating that interspecies variation in a known restriction factor selected for viral counter-adaptations in the context of a documented case of cross-species transmission, our results lend strong support to the evolutionary “arms-race” hypothesis. Importantly, our study confirms that *APOBEC3G* divergence can be a critical determinant of interspecies transmission and emergence of primate lentiviruses, including viruses with the potential to infect and spread in human populations.

## Author Summary

APOBEC3G is a host factor that can inhibit replication of primate lentiviruses, including HIV-1, HIV-2, and the related simian immunodeficiency viruses (SIVs) of African primates. As a consequence, primate lentiviruses encode a protein, called Vif, which can induce degradation of APOBEC3G. Given its antiviral role, APOBEC3G may be an important genetic barrier to interspecies jumping of primate lentiviruses. To study this possibility, we asked whether APOBEC3G affected transmission of SIV from sooty mangabeys (SIVsm) to rhesus macaques and subsequent emergence of pathogenic SIVmac in the 1970s. We found that APOBEC3G of sooty mangabeys and rhesus macaques have divergent protein sequences, and that the Vif proteins of SIVsm (Vif-SIVsm) cannot counteract rhesus macaque APOBEC3G. We mapped Vif-SIVsm resistance to a specific substitution in the N-terminal domain of rhesus APOBEC3G, in which a highly conserved tyrosine is replaced by leucine-arginine (Y→LR). We also identified a viral counter-adaptation, found in the Vif proteins of all SIVmac strains, which specifically confers the ability to antagonize APOBEC3G of rhesus macaques. This change was most likely selected during adaptation of SIV to its new host. Together, these results demonstrate that *APOBEC3G* can serve as a critical genetic determinant of interspecies transmission of primate immunodeficiency viruses.

## Introduction

In addition to the human immunodeficiency viruses (HIV-1 M, N, O and P and HIV-2 A–H), there are more than forty lentiviruses endemic to African old world primates [1], [2]. The distribution of these viruses among modern primates is consistent with a complex history of cross-species transmissions between different host lineages [2], [3]. Well-documented examples include the emergence of both HIV-1 and HIV-2 in humans in the mid-to-late 20^th^ century, and emergence of pathogenic SIVmac in captive Asian macaques in the 1970s [2], [4], [5], [6], [7], [8]. The exact time and circumstances under which SIVsm initially jumped from sooty mangabeys into rhesus macaques to give rise to SIVmac are unknown, but it is very likely that transmission may have resulted from experimental interventions involving the transfusion of material from one species into another [9]. Despite the extraordinary attention the primate lentiviruses have since received from the AIDS research community, very little is known about the impact of host genetic variation on the transmission of these viruses between different primate species, or the degree to which successful emergence of lentiviral pathogens requires adaptation to overcome genetic divergence between reservoir species and newly emergent hosts.

Cellular restriction factors are host factors that render the host cell resistant to viral infection (also referred to as intrinsic immunity) [10], [11]. For many restriction factor genes, the rate of fixation of nonsynonymous changes often exceeds that expected by genetic drift alone, consistent with evolution under strong positive selection, and it is generally assumed that viral pathogens are the source of selective pressure driving evolution of such genes [12], [13], [14]. As a consequence of positive selection, even phylogenetically similar species are likely to differ in terms of restriction factor functionality. Thus, interspecies differences in restriction factor loci could serve as genetic barriers to cross-species transmission and emergence of viruses. One way to test this hypothesis is to focus on known transmission events and ask whether specific restrictions played a role. Viruses that successfully overcome species-level barriers imposed by restriction should harbor adaptive changes (relative to viruses in the established host) that confer resistance to restriction in the new host. Thus, identification of changes which correlate with host-switching events, and which demonstrably overcome restriction(s) imposed by the new host, constitute direct evidence that a specific host restriction factor can act as determinant of cross-species transmission and emergence of viral pathogens. For example, we previously reported that the host restriction factor *TRIM5* can suppress viral replication in vivo, exerting selective pressure during the initial stages of cross-species transmission [15]. More specifically, we demonstrated that allelic variation in *TRIM5* influences the outcome of SIVsm infection in rhesus macaques, and that emergence of SIVsm (as pathogenic SIVmac) required adaptations to overcome the genetic barrier imposed by the most common variant of rhesus macaque TRIM5.

The cellular restriction factor APOBEC3G (apolipoprotein B mRNA-editing enzyme catalytic polypeptide-like 3G) inhibits retroviruses by incorporating into budding virions and inducing hypermutation of the viral cDNA during reverse transcription [16], [17], [18]. *APOBEC3G* (*A3G*) is one of seven members of the *APOBEC3* gene cluster found in primates [19], [20]. A3G is expressed in hematopoietic cell populations, including T cells and myeloid cells, all of which are targets for lentiviral infection [21]. Most lentiviruses counter the antiviral activity of A3G by means of an accessory protein called Vif (viral infectivity factor). Vif functions by bridging A3G proteins with a cellular E3 ubiquitin ligase complex, thus marking A3G for proteasomal degradation [22]. Typically, the interaction between A3G homologs and the Vif proteins of different lentiviruses are species-specific, indicative of Vif-specific adaptation to A3G of the native host [23], [24], [25].

The emergence of SIVmac, and subsequent outbreaks of AIDS in captive macaque colonies in the 1970s, was a striking parallel to the emergence of HIV and outbreaks of AIDS in humans around the same time [5], [26], [27]. To examine the importance of *A3G* as a determinant of cross-species transmission and emergence of primate lentiviruses, we asked whether A3G-mediated restriction influenced the successful transmission of SIVsm from its native host, the sooty mangabey (*Cercocebus atys*), into colonies of Asian rhesus macaques (*Macaca mulatta*). We found that the *A3G* coding sequences of sooty mangabeys and rhesus macaques have several fixed, nonsynonymous differences. Moreover, we found that rhesus A3G (rhA3G) has an unusual polymorphism in the N-terminal domain, in which a highly conserved tyrosine is replaced by a two-amino acid insertion (either Y^59^/LR^59–60^ or Y^59^/LL^59–60^). Structural modeling of the insertion indicates that it is in spatial proximity to residue 128, a previously identified determinant of human and African green monkey A3G sensitivity to the Vif proteins of HIV-1 and SIVagm [24], [28], [29]. The model suggests that these two positions (60 and 128) may define a common Vif-interaction surface on A3G that is exploited by lentiviral Vif proteins.

While we found that Vif from SIVsm can degrade the more ancestral rhA3G^Y^ allele, it was unable to induce degradation of rhA3G^LR^. In contrast, we found that the Vif proteins of multiple, independent SIVmac isolates induced degradation of all three rhesus alleles (rhA3G^LR^, rhA3G^LL^ and rhA3G^Y^). Furthermore, we identified a single ^17^Gly-to-^17^Glu adaptation common to the Vif proteins of multiple SIVmac isolates that confers the ability to degrade rhA3G^LR^ and rhA3G^LL^. This same adaptation occurred at least twice, once during emergence of SIVmac, and again during intentional adaptation of SIVsm to rhesus macaques by experimental passage *in vivo* (SIVsmE543) [30]. Convergent evolution, at a specific Vif residue in at least two independent pathogenic SIVmac viruses, clearly indicates that A3G influenced the emergence of pathogenic SIV and simian AIDS in U.S. macaques. Thus, our results demonstrate that APOBEC3G-mediated restriction initially served as an active barrier to full emergence of SIVsm (as SIVmac) in rhesus macaques, selecting for adaptations rendering the virus insensitive to restriction (and therefore better adapted to the macaque host).

## Results

### Allelic variation in rhesus macaque and sooty mangabey A3G

**Figure 1:**
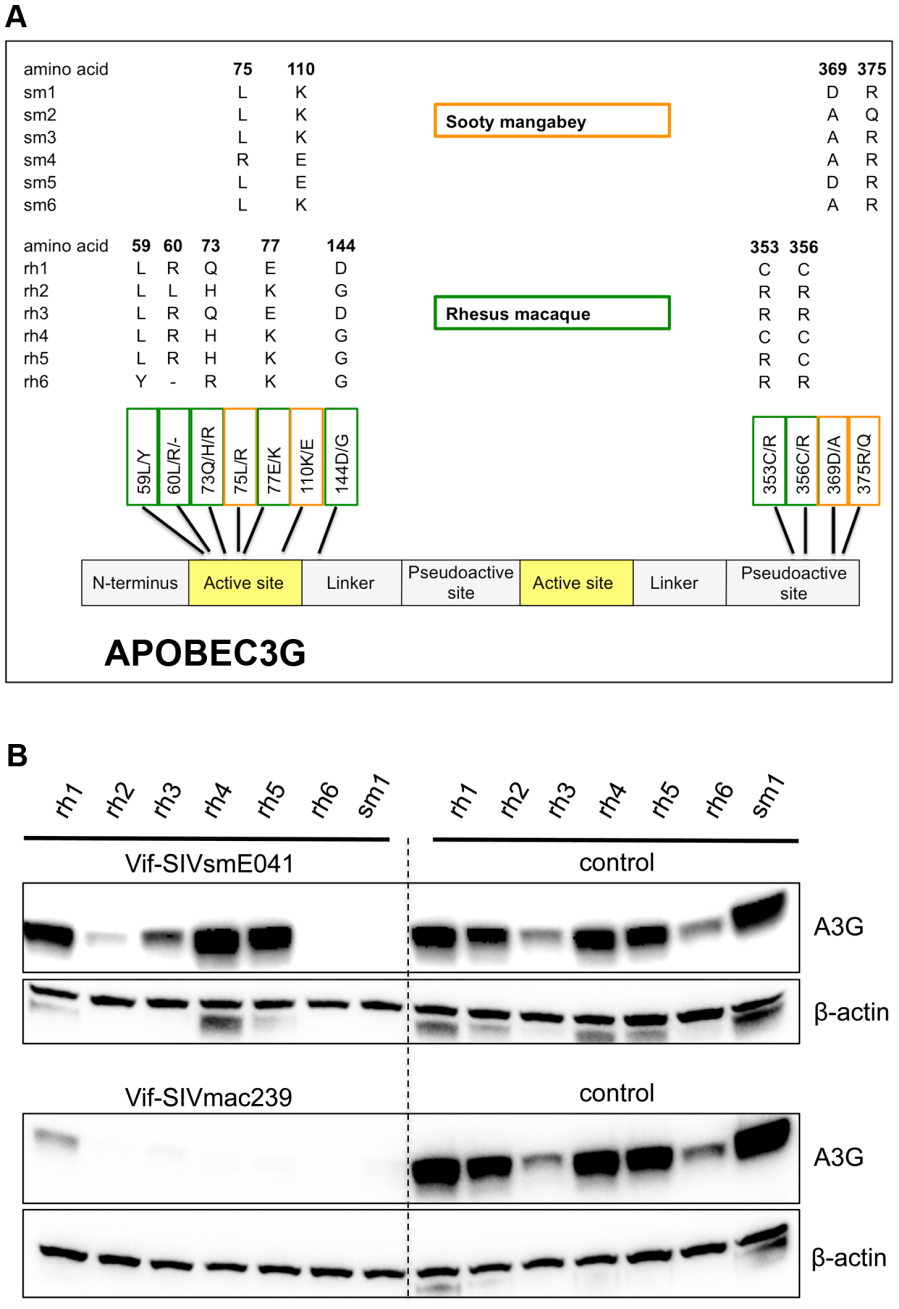
Rhesus macaque and sooty mangabey *A3G* coding sequences are highly polymorphic and differ in sensitivity to Vif-mediated degradation. (A) Shown are nonsynonymous polymorphisms found in cDNA samples from sooty mangabeys (alleles numbered sm1-sm6) and rhesus macaques (alleles numbered rh1-rh6) and their relative distribution across the A3G protein. Sooty mangabey polymorphisms are indicated with orange boxes, rhesus macaque polymorphisms with green boxes. The numbers indicate the position of the amino acid in the protein and the letters indicate the amino acids found at that position. (B) Rhesus macaque and sooty mangabey A3G allele containing plasmids were co-transfected with plasmids expressing Vif-SIVsmE041, Vif-SIVmac239, or an empty vector (no Vif) control (indicated as “control”). The ability of Vif proteins to induce A3G degradation is reflected by a reduced or absent A3G band relative to controls. Anti-β-actin served as a protein loading control. See also Figures S1, S2 and S3.

To investigate the possible role of A3G as a genetic barrier to transmission of SIVsm from its natural host into rhesus macaques, we first surveyed cDNA samples from multiple individual sooty mangabeys and rhesus macaques for polymorphisms in the *A3G* coding sequence. We identified seven nonsynonymous single-nucleotide polymorphisms (nsSNPs) in rhesus macaque *A3G* (rh*A3G*) and four nsSNPs in sooty mangabey *A3G* (sm*A3G*) (Figure 1A, see also Figure S1). Changes were only chosen when they were detected in at least three independent clones. With one exception (144D/G), the nsSNPs clustered either in the first active domain or the second pseudoactive domain of A3G. In addition to single amino-acid substitutions (59Y/L, 73Q/H/R, 77E/K, 144D/G, 353C/R and 356 C/R), rhesus macaques also have an insertion at position 60 (L or R). The four substitutions in sooty mangabeys included two in the first active domain (76L/R and 111K/E) and two in the second pseudoactive domain (370D/A and 376R/Q). It is notable that polymorphisms in both species are clustered in the same two regions of the protein, and that almost all substitutions in both regions resulted in a difference in charge (either a charge switch or gain/loss of a charge). It is striking that polymorphism at some of these sites occur in multiple species; for example, one of the polymorphic sites found in rhesus macaque (residue 77) and one found in sooty mangabey (residue 110) are both polymorphic in African green monkeys [31]. Similarly, position 130 is polymorphic in both rhesus macaques (either asparagine or aspartic acid) and African green monkeys (either aspartic acid or histidine) [32]. The clustering of nonsynonymous polymorphisms in the first active domain, including polymorphisms at sites that are polymorphic in more than one species, is consistent with a region undergoing extensive, adaptive evolution in response to positive selection. We sought to determine whether any of these variants had functional consequences with respect to restriction of SIVsm and SIVmac.

### Multiple rhesus macaque A3G alleles resist degradation by Vif-SIVsm

We tested all of the rhesus macaque A3G alleles (rh1-rh6) and sooty mangabey A3G alleles (sm1-sm6) for incorporation into SIVmac239ΔVif virions and for the ability to restrict SIVmac239ΔVif (Figure S2). We found that all twelve alleles (six sooty mangabey and six rhesus macaque) are incorporated into budding virions in the absence of Vif, and all twelve were able to restrict infectivity. Thus, the differences in protein sequence that distinguish the alleles do not result in substantial defects in overall A3G function.

We then asked whether variation in rhesus macaque APOBEC3G results in differences in resistance or sensitivity to degradation by lentiviral Vif proteins. Specifically, we tested the Vif proteins of two viruses, a primary sooty mangabey isolate (SIVsmE041) and a pathogenic, macaque-adapted isolate (SIVmac239), for the ability to induce degradation of each of the rhA3G alleles (Figure 1B). Of the six different rhA3G alleles tested, only one (allele rh6) was clearly sensitive to degradation by Vif-SIVsmE041, although one other allele (rh2) was weakly sensitive to degradation in the presence of Vif-SIVsmE041. In contrast, all six rhesus macaque alleles (rh1-rh6) were clearly degraded by Vif-SIVmac239. Both Vif-SIVsmE041 and Vif-SIVmac239 induced degradation of all six sooty mangabey A3G alleles (Figure 1B and Figure S3).

### LR^59–60^ renders A3G resistant to Vif-SIVsmE041 induced degradation and is unique to macaques

**Figure 2:**
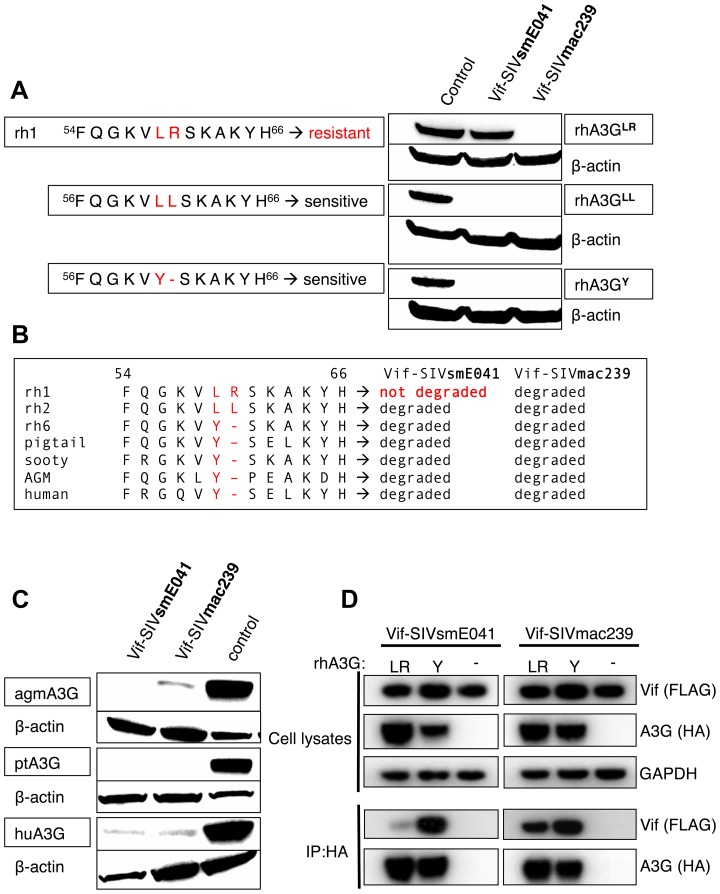
A Tyr to Leu-Arg substitution at position 59/60 makes rhA3G resistant to Vif-SIVsmE041 induced degradation. (A) Site-directed mutagenesis was used to mutate a resistant rhesus macaque allele (rh1) to either one of two naturally occurring sensitive alleles (rhA3G^LL^, rhA3G^Y^), their ability to resist Vif-mediated degradation was visualized by western blot. We used an empty vector control to test expression in the absence of Vif (indicated as “control”). Anti-β-actin served as a protein loading control. (B) Partial alignment of A3G proteins from different species (rh1, rh2, rh6 = rhesus macaque alleles; pigtail = pigtail macaque, sooty = sooty mangabey, AGM = African green monkey). Also indicated is the observed sensitivity of the A3Gs (listed as “not degraded” or “degraded”) to either Vif-SIVsmE041 or Vif-SIVmac239 induced degradation. (C) Immunoblot showing activity of Vif-SIVsmE041, Vif-SIVmac239 and an empty vector (no Vif) control against A3G proteins from African green monkey (Haplotype 1 see [31]), pigtail macaque and human. Equal expression levels were confirmed using anti-β-actin as a loading control. (D) Co-immunoprecipitation experiment with Vif-SIVmac239 and Vif-SIVsmE041 and rhA3G^LR^, rhA3G^Y^ or a no A3G control. Whole-cell lysates were analyzed by immunoblotting using antibodies specific for FLAG (Vif), HA (A3G) or GAPDH (loading control). The immunoprecipitations (IP) were analyzed with antibodies specific for FLAG (Vif) or HA (A3G).

All of the rhesus A3G alleles that resist Vif-SIVsmE041-mediated degradation have a Tyr-to-Leu substitution at position 59, followed by either an Arg insertion (alleles rh1, rh3, rh4, and rh5), or a Leu insertion (allele rh2). In contrast, the rh6 allele and all six of the sooty mangabey alleles retain a highly conserved Tyr at position 59, and lack any additional insertions. Importantly, the Y/LR and Y/LL substitutions are the only differences that distinguish all of the Vif-SIVsmE041-resistant A3G proteins from all of the sensitive alleles, leading us to hypothesize that these substitution are responsible for the observed differences in sensitivity to degradation by Vif-SIVsmE041. To test this hypothesis, we used site-directed mutagenesis to change one of the resistant alleles (rh1) at position 59/60, either substituting the Arg for an Leu (LR → LL) or removing the insertion and reverting position 59 to the ancestral/conserved Tyr (LR → Y). Both mutations (R60L and LR59/60Y) rendered rhA3G sensitive to Vif-SIVsmE041 degradation, but did not alter sensitivity to Vif-SIVmac239-mediated degradation (Figure 2A).

Notably, the tyrosine at position 59 is highly conserved, and is found at the homologous position in most other primate A3G proteins, whereas the additional insertions (LR and LL) are unique to Asian macaques (Figure 2B). We therefore generated A3G expression constructs representing three additional primate species, including pigtail macaque (*Macaca nemestrina*), African green monkey (*Clorocebus aethiops*) and human (*Homo sapiens*), and tested these for sensitivity or resistance to degradation by Vif-SIVsmE041 and Vif-SIVmac239 (Figure 2C). All three A3G orthologs retain the ancestrally conserved Tyr59, and in accordance with our earlier observations, all three of these were degraded by expression of Vif-SIVsmE041 and Vif-SIVmac239.

Finally, we asked whether the Y/LR substitution had a direct effect on the Vif-A3G interaction, using co-immunoprecipitation (Figure 2D). We compared binding of rhA3G^LR^ and rhA3G^Y^ to both Vif-SIVmac239 and Vif-SIVsmE041. While Vif-SIVmac239 bound equally well to both rhA3G^LR^ and rhA3G^Y^, there was a dramatic reduction in binding of Vif-SIVsmE041 to rhA3G^LR^ relative to rhA3G^Y^. This result suggests that the Y/LR substitution exerts a direct effect on the Vif-A3G interaction.

### Position 130 can influence sensitivity of rhA3GLR to Vif-SIVsmE041

Compton and Emerman previously described a N/D polymorphism at position 130 in rhA3G [32]. Out of 219 rhesus macaques that we screened, almost half carried an asparagine at position 130 (animals with the 130N/N or 130N/D genotypes) (Figure S4B), and alleles rh1-rh6 all have an asparagine at position 130. Strikingly, of 72 animals that were homozygous for the 60LR allele (LR/LR genotype), only three had an aspartic acid at position 130 (LR/LR + N/D genotype) (Figure S4C). There were no LR/LR + D/D homozygous animals. In contrast, 69 animals were of the 60LR/LR 130N/N genotype (95.8%). Thus, despite the high frequency of both 60LR and 130D, the combined 60LR+130D haplotype was present at very low frequency (Figure S4C).

Because the 60LR + 130D haplotype appears to be rare in our cohort, we decided to test the functional consequences of the residue at position 130 in the context of alleles differing at position 59/60. To do this, we constructed a series of double mutants by site directed mutagenesis (Figure S4A). We found that rhA3G^LR^, rhA3G^LL^ and rhA3G^Y^ remained sensitive to Vif-SIVmac239 regardless of the presence of an asparagine or aspartic acid at position 130. Likewise, degradation of rhA3G^LL^ and rhA3G^Y^ by Vif-SIVsmE041 was also unaffected by the substitution at position 130. Surprisingly, however, introduction of an asparagine at position 130 rendered rhA3G^LR^ sensitive to degradation by Vif-SIVsmE041. Given the rarity of the 60LR+130D haplotype, it is tempting to speculate that the low frequency of this allele reflects the history of SIV outbreaks in captive macaque colonies. However, proving or disproving this possibility is complicated by the use of selective breeding procedures in U.S. primate research centers and a current lack of information regarding allele frequencies in natural macaque populations.

### Frequency of rhA3G^LR^ in rhesus macaques

To determine the respective frequencies of the rhA3G^LR^, rhA3G^LL^ and rhA3G^Y^ alleles, we used archived genomic DNA samples to genotype 219 captive-bred rhesus macaques (Table 1). The allelic frequency of rhA3G^LR^ was an impressive 48.9%, followed by rhA3G^Y^ (24.7%) and rhA3G^LL^ (26.5%). Likewise, homozygote genotype frequencies were the lowest for rhA3G^Y/Y^ and rhA3G^LL/LL^ (8.7% and 14.6%, respectively) and highest for rhA3G^LR/LR^ (32.9%). Using BLAST and sequence alignments, we identified the presence of the LR insertion in at least one additional species of Asian macaques, *Macaca fasicularis* (crab-eating macaque) (see also [33]). We confirmed this finding by genotyping genomic DNA from 17 crab-eating macaques, and found that this species also harbors the Y/LR polymorphism at positions 59–60 (genotype frequencies in this small sampling were: 23% LR/LR; 42% Y/Y; 35% Y/LR). Thus, the A3G^LR^ allele likely dates back at least as far as the common ancestor of rhesus macaques and crab-eating macaques, giving a minimum age of approximately 1.2–2.5 million years ago [34]. So far, all other primate A3G sequences in the public sequence databases have the ancestral Tyr at position 59, except for *Colobinae* spp. which have a Ser at that position [32]. Interestingly, Compton and Emerman also describe a three amino-acid insertion in *Colobinae* A3G four residues downstream (positions 64–66) that affects sensitivity to degradation by Vif from SIVagm.Sab in tissue culture.

**Table 1:** Rhesus macaque *APOBEC3G* genotype frequencies.

Genotype	Number of Animals (n = 219)	Frequencies %
**Y/Y**	19	8.7
**LL/LL**	32	14.6
**LR/LR**	72	32.9
**Y/LL**	26	11.9
**Y/LR**	44	20
**LL/LR**	26	11.9

### Amino acid 17 in the N-terminal half of Vif governs the interaction with rhA3G^LR^

**Figure 3:**
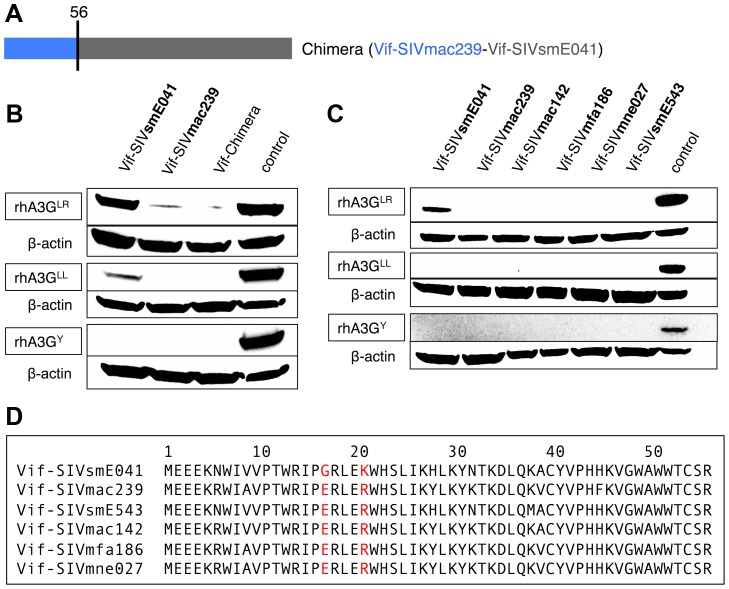
The determinant in Vif for interaction with rhA3G^LR^ lies within the first 56 amino acids of the Vif protein. (A) Cartoon of a chimeric protein containing the first 56 amino acids of Vif-SIVmac239 and the rest of Vif-SIVsmE041. (B) Immunoblot showing activity of the parental Vif proteins (Vif-SIVsmE041 and Vif-SIVmac239), the Vif-Chimera and an empty vector (no Vif) control against the three different rhA3G alleles (rhA3G^LR^, rhA3G^LL^ and rhA3G^Y^). Anti-β-actin served as a protein loading control. (C) Immunoblot showing activity of six Vif proteins and an empty vector (no Vif) control against the three rhA3G alleles (rhA3G^LR^, rhA3G^LL^ and rhA3G^Y^). Anti-β-actin served as a protein loading control. (D) Partial alignment of the NTD of the Vif protein from six different SIV strains as indicated. Highlighted in red are amino acids 17 and 21 which are conserved amongst Vif proteins from macaque derived SIV strains but differ in Vif from SIVsmE041. See also Figure S5.

We next sought to identify adaptations in Vif-SIVmac239 that conferred the ability to degrade rhA3G^LR^. Previous studies reported that sequences in the N-terminal part of Vif proteins are important for determining the species-specificity of Vif interactions with A3G [35], [36], [37], [38]. Because there are multiple differences in the N-terminal regions of the Vif-SIVsmE041 and Vif-SIVmac239 proteins, we first constructed a chimeric Vif protein in which the N-terminal 56 residues of Vif-SIVsmE041 were replaced with the homologous portion of Vif-SIVmac239 (Figure 3A). As with Vif-SIVmac239, expression of the chimeric Vif protein was also able to induce degradation of all three rhA3G alleles (Figure 3B), indicating that adaptative changes to overcome rhA3G-mediated restriction might be found within the first 56 amino acids of the Vif protein.

We tested a series of additional Vif proteins derived from SIV isolates of several different host species, including rhesus macaque-, pig-tailed macaque-, cynomolgus macaque- and sooty mangabey-derived isolates. These included Vif from SIVsmE543, a virus isolated after experimental passage of SIVsm through two rhesus macaques [27], [30]; Vif from SIVmac142, which was isolated from a rhesus macaque infected unintentionally *in utero* [39]; Vif from SIVmne027, which was isolated from a pig-tailed macaque [27]; and Vif from SIVmfa186, a virus isolated from the lymph node of an SIV+ cynomolgus macaque (in vivo passage history unknown) [40], [41]. All four SIV Vifs (Vif-SIVsmE543, Vif-SIVmac142, Vif-SIVmne and SIVmfa186) were able to induce degradation of all three rhA3G variants (Figure 3C). In contrast, and as shown previously, Vif-SIVsmE041 tested in parallel induced degradation of rhA3G^Y^ and rhA3G^LL^, but not of rhA3G^LR^.

To rule out the possibility that Vif-SIVsmE041 is unique among SIVsm viruses, we also tested the Vif protein of a second primary SIVsm isolate (SIVsmCFU212, GenBank Accesssion #JX860407) [42], as well as the Vif proteins of two SIVsm isolates that were each derived by infection of single pig-tailed macaques with primary SIVsm (SIVsmPBj and SIVsm-PG) [27] [43]. As with Vif-SIVsmE041, the Vif-SIVsmCFU212, Vif-SIVsmPBj and Vif-SIVsmPG proteins all failed to induce degradation of rhA3G^LR^ but were able to induce degradation of rhA3G^Y^ (Figure S6).

**Figure 4:**
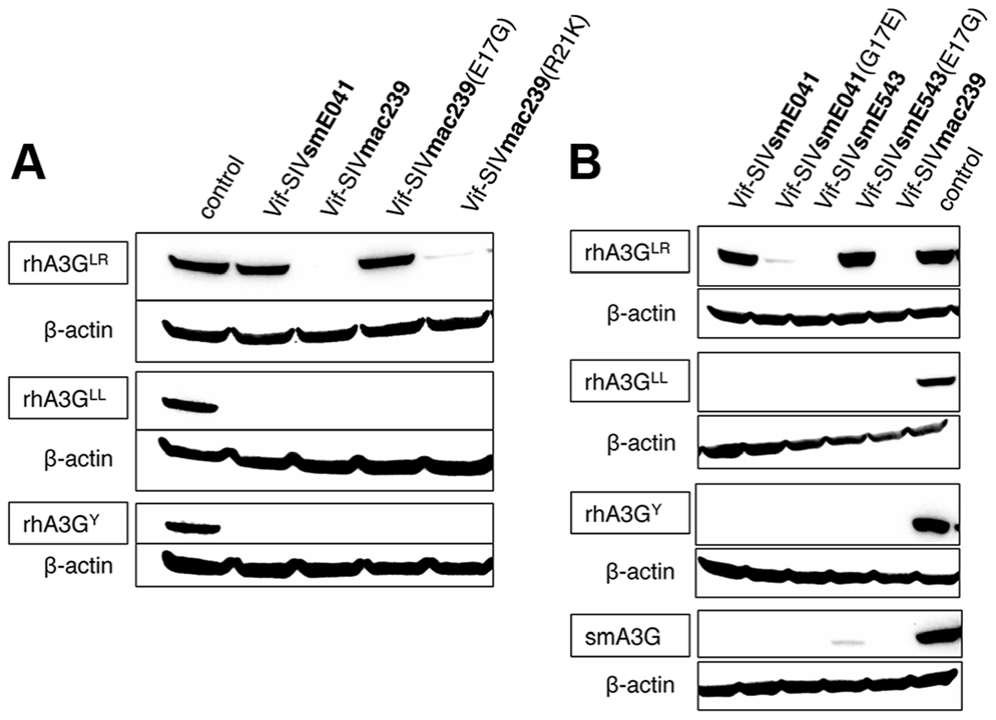
The negatively charged glutamic acid at position 17 in the primate Vif protein allows for interaction with rhA3G^LR^. (A) Immunoblot showing activity of Vif-SIVsmE041, Vif-SIVmac239, the mutants Vif-SIVmac239(E17G) and Vif-SIVmac239(R21K), and an empty vector (no Vif) control against the three rhA3G alleles (rhA3G^LR^, rhA3G^LL^ and rhA3G^Y^). Anti-β-actin served as a protein loading control. (B) Immunoblot showing activity of Vif-SIVsmE041 and Vif-SIVsmE543 and their mutants (Vif-SIVsmE041(G17E), Vif-SIVsmE543(E17)) as well as Vif-SIVmac239 and an empty vector (no Vif) control against the three rhA3G alleles and a sooty mangabey A3G. Anti-β-actin served as a protein loading control.

By inspecting an alignment of the first 56 amino acids of Vif from multiple SIVmac and SIVsm isolates, we identified two residues (positions 17 and 21) that were conserved amongst all SIVmac strains and SIVsmE543 yet differed from the Vif proteins of SIVsm viruses (Figure 3D and Figure S5). We therefore substituted the individual residues at positions 17 and 21 in Vif-SIVmac239 with the corresponding residues of Vif-SIVsmE041, thereby changing the negatively-charged glutamic acid to an uncharged glycine (E17G) or the positively-charged arginine to a positively charged lysine (R21K), and asked whether either substitution had an effect on the ability of Vif-SIVmac239 to degrade rhA3G^LR^. We tested both mutants for the ability to induce degradation of rhA3G^LR^, rhA3G^LL^ and rhA3G^Y^ by co-transfection (Figure 4A). Expression of mutant Vif-SIVmac239(E17G), in which the negatively charged amino acid was changed to an uncharged amino acid, failed to induce degradation of rhA3G^LR^, but was nonetheless functionally intact, as evidenced by the ability to degrade both the rhA3G^LL^ and rhA3G^Y^ proteins.

We also altered the Vif of rhesus-macaque isolate SIVsmE543 at the same position, again changing the glutamic acid to a glycine, and tested the mutant against the three rhA3G alleles and a sooty mangabey allele (Figure 4B). As with Vif-SIVmac, as a consequence of changing this one amino acid, the Vif-SIVsmE543 protein was unable to induce degradation of rhA3G^LR^, yet retained the ability to induce degradation of rhA3G^LL^, rhA3G^Y^ and smA3G.

To confirm that this loss of Vif function (the ability to degrade A3G) was due to the amino acid at position 17, we also performed the converse experiment, altering Vif-SIVsmE041 by mutation of residue 17 from a glycine to a glutamic acid, and testing the mutant against the three rhA3G alleles (Figure 4B). We observed that the Vif-SIVsmE041(G17E) mutant gained the ability to degrade the rhA3G^LR^ allele, while also retaining the ability to induce degradation of the other two rhA3G alleles (as well as sooty mangabey A3G).

To further confirm the importance of the residue at position 17, we looked at additional Vif sequences from multiple, previously described SIVsm strains [42] (including SIVsmCFU212, SIVsmPBj and SIVsm-PG) and found that they all had a glycine at position 17 (Figure S5 and Figure S6). Likewise, HIV-2 Vif and Vif-SIVstm (an SIV from stump tailed macaques, or *Macaca arctoides*), both of which represent independent cross-species transmissions originating from SIVsm, also have a glycine at position 17. Thus, Gly17 is well conserved among SIVsm lineages, whereas Glu17 is very likely to have been evolutionarily derived in conjunction with emergence of SIVmac in rhesus macaques, and independently during experimental adaptation of SIVsmE543 to rhesus macaques. This observation, coupled with our experimental results, strongly suggests that the G17E substitution found in viruses of the SIVmac lineage represents adaptation to overcome the interspecies genetic barrier imposed by rhA3G^LR^ alleles.

### The Vif-SIVmac239(E17G) mutation results in increased incorporation of rhA3G^LR^ into virions and lower infectivity

**Figure 5:**
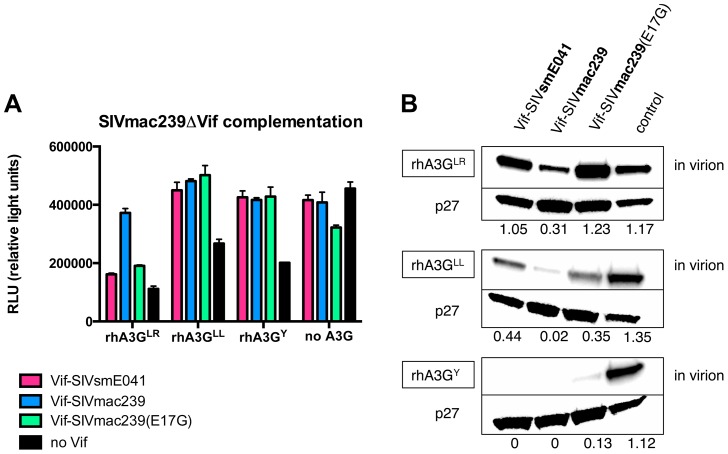
Mutating the negatively charged glutamic acid at position 17 to glycine in Vif-SIVmac239 reduces infectivity and increases incorporation of rhA3G^LR^ into virions. (A) Shown is one representative of two independent infectivity assay experiments. Infectivity was measured by titration on TZM-bl indicator cells. The virus was produced in HEK-293T/17 cells by co-transfection of a full-length SIVmac239ΔVif plasmid, a plasmid coding for one of the three A3G alleles (rhA3G^LR^, rhA3G^LL^ or rhA3G^Y^) or a no A3G control and a plasmid coding for one of three Vif proteins (Vif-SIVsmE041, Vif-SIVmac239 or Vif-SIVmac239(E17G)) or a no Vif, empty vector control. Error bars indicate the standard deviation of three replicate infections. (B) Immunoblot was used to measure packaging of rhA3G into virions using virus preparations also used in the above infectivity experiment. The amount of virus present is measured by detecting the SIV p27 core antigen with an anit-p27 antibody. The relative density of each A3G band (as compared to the corresponding p27 band) is indicated below each lane.

We next tested the effects of Vif-SIVsmE041, Vif-SIVmac239 and the Vif-SIVmac239(E17G) mutant in an infectivity assay. To this end, we trans-complemented an SIVmac239 Vif deletion mutant (SIVmac239ΔVif) by co-expression of Vif-SIVsmE041, Vif-SIVmac239 or the Vif-SIVmac239(E17G) mutant, and asked whether these rescued viral infectivity in the presence of different rhA3G alleles. Specifically, complementation with the Vif-SIVmac239(E17G) mutant or with Vif-SIVsmE041 resulted in reduced infectivity relative to virus complemented with wild type Vif-SIVmac239 in the presence of rhA3G^LR^ (*p*-value<0.0001 for both Vif-SIVmac239(E17G) vs. Vif-SIVmac239 and Vif-SIVsmE041 vs. Vif-SIVmac239). There was no significant difference between the Vif-SIVmac239(E17G) and Vif-SIVsmE041 complemented viruses, relative to rhA3G^LR^ (Figure 5A). In contrast, both proteins were nearly as effective as wild type Vif-SIVmac239 in the presence of the other rhesus alleles (rhA3G^LL^ and rhA3G^Y^). Importantly, the observed activity of the different Vif proteins against the rhA3G alleles mirrored the results obtained from degradation experiments (compare Figure 5A and Figure 4).

In parallel, we analyzed the levels of encapsidated rhA3G in the same virion preparations used for the infectivity assays, by immunoblotting and densitometry (Figure 5B). In accordance with the degradation and infectivity experiments, we observed reduced incorporation of rhA3G^LR^ in Vif-SIVmac239 complemented virions relative to virions complemented with Vif-SIVsmE041 (3.4-fold increase relative to p27) or Vif-SIVmac239(E17G) (4-fold increase relative to p27). Incorporation of rhA3G^Y^ in the presence of any of the three Vif proteins was essentially undetectable. Similar to the results of degradation experiments (Figure 1B and Figure 3B), there were low but detectable levels of rhA3G^LL^ incorporation into Vif-SIVsmE041-complemented and Vif-SIVmac239(E17G)-complemented virions, and even very low levels of rhA3G^LL^ incorporation into Vif-SIVmac239 complemented virions. Thus, it is possible that rhA3G^LL^ also confers some resistance to Vif, albeit significantly less than rhA3G^LR^.

## Discussion

New viral pathogens arise when viruses succeed in jumping from natural reservoirs into new host populations, resulting in emerging infectious diseases such as AIDS, SARS and pandemic influenza [6], [44], [45], [46]. The importance of ecological factors in viral emergence is well documented, whereas the significance of host genetic differences as barriers to emergence is still not clear [47]. In particular, it is believed that cellular antiviral restriction factors can act as blockades to cross-species transmission and viral emergence, yet very few studies have examined this possibility *in vivo*. The emergence of SIVmac and outbreaks of simian AIDS in outbred colonies of macaques in the 1970s provides an unusual opportunity to examine the impact of specific, host-encoded restriction factors in the context of an emerging pathogen. Focusing on SIVsm and SIVmac has multiple advantages for understanding the genetics of lentiviral emergence: both the natural reservoir host and the emergent new host are known (sooty mangabeys and rhesus macaques, respectively); archived samples and cloned, primary viral isolates are readily available; and most importantly, several key host-encoded restriction factors that target primate lentiviruses are known and have been well-characterized at the molecular level [48], [49], [50], [51].

Cellular restriction factors that target replication of primate lentiviruses include A3G [17], TRIM5α [52], BST2/Tetherin [53], [54], [55] and SAMHD1 [56], [57]. Here, we demonstrate that spread of SIVsm in rhesus macaques and emergence of pathogenic SIVmac and simian AIDS required viral adaptation to overcome restriction by rhesus macaque *A3G*. In particular, we found that *A3G* is polymorphic in rhesus macaques, and that the most frequent rhesus A3G alleles have complex substitutions in the N-terminal domain (Y^59^/LR^59–60^ and Y^59^/LL^59–60^). We found that Vif from SIVsm, a virus that jumped species at least twice (to emerge as SIVmac in macaques and as HIV-2 in humans) does not degrade the most common rhesus allele (rhA3G^LR^), whereas SIVmac Vif degrades all three classes of rhesus alleles (rhA3G^LR^, rhA3G^LL^ and rhA3G^Y^).

**Figure 6:**
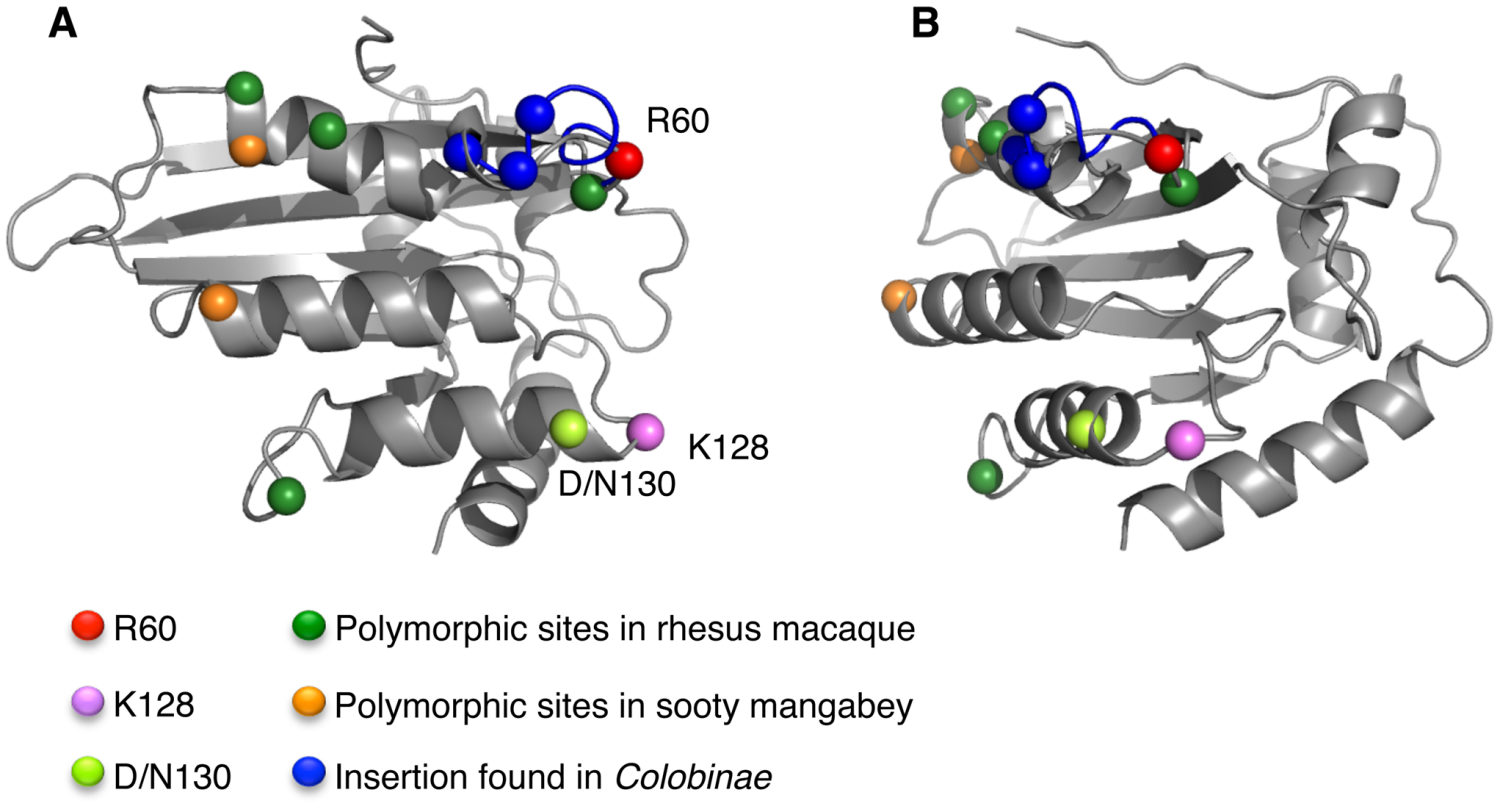
Model of the N-terminal domain of rhesus APOBEC3G. A model of the N-terminal domain of rhA3G^LR^ was generated using the PHYRE2 server [59] using the A3C structure PDB: 3VOW as a model. (A) Position 60 is shown as a red sphere. A previously identified site of positive selection and a determinant of species tropism, K128, is shown as a violet sphere. These two independent determinants of species tropism are predicted to occur on the same face of the A3G N-terminal domain. The site of another previously identified polymorphism of rhesus macaques [32], D/N130, is shown as a light green sphere. Indicated in blue is an insertion found in *Colobinae* monkeys [32]. Measurements using the predicted Cα backbone indicate that these residues are within 20 Å of each other. Additional A3G polymorphisms identified in this study within rhesus macaques (green spheres) or sooty mangabeys (orange spheres) are shown. (B) The same structure as in A, rotated by 270° around the Y-axis.

So far, two important determinants (amino acids 128 and 130) in A3G have been shown to play a role in conferring species-specific sensitivity to Vif [24], [25], [28], [29], [31], [32], [35] suggesting that these residues might be part of a Vif-binding domain. At present, there are no structural data for the N-terminal active domain of A3G. To visualize how the rhesus ^59^Y/^59^LL^60^/^59^LR^60^ polymorphism might also affect interactions with Vif proteins, we modeled the N-terminal sequence of rhA3G onto the recently published human A3C crystal structure [58], [59] (Figure 6). Unlike A3G, A3C has only one active domain; however, both the sequence and predicted secondary structure of A3C are similar to the N-terminal active domain of A3G, such that the structure is a useful approximation [60].

Using this hypothetical model of the N-terminal active site of rhA3G^LR^, we compared the predicted positions of residues which are known or thought to influence species-specific interactions of A3G and Vif, including Arg60 (identified in this study), Lys128 [24], [28] and Asp/Asn130 [32]. The model suggests that Arg60 and Lys128 are probably located on the same face of A3G, within approximately 20 Å of each other. Because variation at both sites affects Vif-mediated degradation, the simplest explanation is that residues 60 and 128 are part of a single Vif-binding surface on A3G (this does not exclude the possibility that different Vif homologs have evolved to make different contacts with the same surface). Similarly, an insertion at position 64–66 in *Colobinae* A3G (indicated in blue spheres in Figure 6) can also affect degradation by Vif proteins of primate lentiviruses [32]. It would therefore appear that these three documented determinants of species-specific antiviral activity might occur on a single face of the A3G protein. This observation offers indirect but compelling evidence that divergent lentiviral Vif proteins have exploited the same or closely overlapping binding sites on primate A3G.

The adaptation of SIVmac to rhesus macaques included a G17E substitution in Vif. Vif proteins from all SIVmac isolates tested degraded rhA3G^LR^ and all carried the G17E change, including Vif from the independently derived SIVsmE543 - a clear case of convergent evolution in a viral accessory protein. Our results establish that interspecies variation in cellular restriction factor A3G can pose a selective barrier to emergence of AIDS-causing viruses, and just as adaptation of Vif contributed to the emergence of pathogenic SIVmac, Vif plasticity could have contributed to the modern distribution of simian immunodeficiency viruses among their respective primate reservoirs.

The means by which SIVsm was initially transmitted to rhesus macaques in US primate research centers is not known, although indirect evidence suggests it was the unintentional result of experimental exchange of infected material between the two species in the 1960s or early 1970s [9]. Thus, the original transmission event(s) may not recapitulate the kinds of mechanisms that are thought to typically give rise to interhost transmission of primate retroviruses in nature, such as biting, fighting with, or preying on, viremic individuals. Nevertheless, replication of primary isolates of SIVsmm in rhesus macaques is highly variable and typically much lower relative to SIVmac isolates, such that the emergence of SIVmac is very likely to have required adaptations to overcome genetic divergence between the reservoir (sooty mangabey) and spillover (rhesus macaque) hosts [15], [61], [62] Our data indicate that adaptation to overcome restriction by a specific subset of rhesus macaque A3G alleles played a key role in spread of SIVsm in captive colonies of rhesus macaques, ultimately contributing to its emergence as pathogenic SIVmac. At the time of initial cross-species exposure, SIVsm was most likely sensitive to rhA3G alleles with an LR at position 59/60 (and perhaps to a lesser extent, alleles with an LL at position 59/60), but not to alleles with a Y at position 59.

It is possible that animals bearing rhA3G^Y^ alleles served as evolutionary “stepping-stones”, by allowing for higher levels of replication and higher probability of continued transmission and spread of SIVsm in the captive rhesus macaque population. The G17E mutation in Vif, whether it coincided with the initial cross-species transmission or appeared subsequently, would have allowed the virus to spread to a larger percentage of the macaque population (e.g., those bearing restrictive A3G^LR^ alleles), fostering further adaptation to the new host.

It is tempting to speculate that the current frequency of rhA3G^LR^ alleles in U.S. macaque colonies was directly influenced by the emergence of SIVmac and outbreaks of simian AIDS in the 1970s and early 1980s. However, the high frequency of rhA3G^LR^ in macaque colonies should also reflect the influence of founder effects, depending on the initial frequency of the allele in the founder populations that were used to establish and maintain breeding colonies at the National Primate Research Centers (NPRC). Because selective breeding programs are used to intentionally maintain diversity in the NPRC colonies, deviations from Hardy-Weinberg equilibrium cannot reliably be used to estimate the relative contributions of selection and founder effects. In order to study the possible influence of SIV emergence on host *A3G* allele frequencies, it may be necessary to recover archived genomic DNA samples from that era, if possible, and to compare historical allele frequencies of captive macaques to allele frequencies of wild macaques in Asia.

At least one study describes analysis of A3G polymorphism in a cohort of SIV-infected macaques, and reports a weak, possible association between rhA3G variation and viral replication [63]. Importantly, the virus in that retrospective study was SIVmac239, a virus that is already well-adapted to the rhesus macaque host (and has been used extensively as a model for preclinical AIDS research for almost 30 years) [39], [64], [65], [66], [67]. Thus, the modest correlation reported by Weiler *et al.* is consistent with our observation that Vif-SIVmac can induce degradation of all three allelic forms of rhA3G, and should be largely unaffected by the A3G genotype.

We have also reported that variation in another restriction factor, TRIM5, correlates with large differences in viremia in rhesus macaques infected with SIVsmE543, and that TRIM5-mediated suppression selects for emergence of resistant SIVsmm variants [15]. As with A3G and SIVmac239, the impact of rhesus TRIM5 on replication of established SIVmac isolates is minimal [63], [68]. Thus, our data and previously published studies of both A3G and TRIM5 in macaques support the hypothesis that restriction has its greatest biological impact as a modulator of interspecies transmission and emergence of viral pathogens. Evidence of positive selection in primate restriction factor loci may therefore reflect recurrent or continuous lineage-specific exposures to viruses of other species, with repeated transmission events and outbreaks of disease driving fixation of resistance alleles. This could occur in situations where different host populations come into physical contact - for example, as the result of fighting over territory or when one species routinely preys on another. Whether restriction factors continue to play a role after a pathogen becomes established in the new host is not as clear, and will require further study.

## Materials and Methods

### Cell lines

Human Embryonic Kidney 293T/17 (HEK293T/17) cells were obtained from American Type Culture Collection (Manassas, VA). HeLa Human cervical carcinoma (TZM-bl) cells were obtained through the NIH AIDS Reagent Program, Division of AIDS, NIAID, NIH: TZM-bl from Dr. John C. Kappes, Dr. Xiaoyun Wu and Tranzyme Inc. [69], [70], [71], [72]. Both cell lines were grown in Dulbecco's modified Eagles medium (DMEM) containing 10% fetal bovine serum (FBS) (Invitrogen; Carlsbad, CA)

### Immunoblotting

HEK293T/17 cells were seeded at a density of 6×10^5^ cells per well in a 6-well plate or at 2×10^6^ in a 60 mm dish or a T25 flask. Cells were co-transfected with appropriate amounts of the indicated plasmids using either the GenJet transfection system (SignaGen; Ijamsville, MD) or the Lipofectamine Plus reagent (Invitrogen; Carlsbad, CA) following the manufacturer's recommendations and harvested 48–72 hours post transfection. Cells were lysed in M-PER reagent (Pierce Biotechnology, Rockford, IL) or Pierce IP-lysis Buffer (Thermo Fisher Scientific, Waltham, MA), mixed with an equal volume of 2× laemmli sample buffer (Sigma, St. Louis, MO) and solubilized by boiling for 10 min at 99°C. Protein was separated by SDS/PAGE and tagged proteins were detected with either mouse monoclonal anti-V5 antibody (Invitrogen; Carlsbad, CA) or mouse monoclonal anti-HA antibody (Covance; Dedham, MA) using dilutions recommended by the manufacturer. β-actin was detected with mouse monoclonal beta actin-HRP antibody at a concentration of 1∶1000 (Abcam Inc., Cambridge, MA). Virus was harvested by centrifugation (see “Encapsidation of Virus” section). Lysed, replication-competent virus was detected with monoclonal SIVmac anti-p27 Antibody (NIH AIDS Reagent Program) [73] at a dilution of 1∶2000.

### Co-immuno-precipitation

FLAG-tagged Vif expression plasmids (100 ng) were co-transfected with HA-tagged A3G expression plasmid (900 ng) and 1000 ng GST-expressing filler plasmid in 293T cells in a 6-well format (2 µg DNA total). Cells were lysed two days post transfection in a mild lysis buffer (0.5% triton X-100 in 1× PBS supplemented with EDTA-free protease inhibitor cocktail, Roche) on ice and the cell lysates were cleared by centrifuging at 14,000×g for 10 minutes at 4°C. Cleared lysates were incubated with EZ-View anti-HA beads (Sigma) at 4°C for two hours. Beads were washed 4 times in cold mild lysis buffer, followed by 4 additional washes in cold stringent lysis buffer (1% triton, 0.1% SDS, 500 mM NaCl in PBS). Proteins were eluted from the beads by boiling in LDS loading buffer (Sigma). Proteins were analyzed by western blot for Vif (FLAG), A3G (HA) and tubulin.

### Viruses

Full-length SIV viruses were produced in HEK293T/17 cells by transfection using the GenJet transfection system (SignaGen; Ijamsville, MD). SIVmac239ΔVif [74] plasmids were co-transfected with 4 µg pcDNA3.1-Vif.HA (or and empty vector control) and 3 µg pcDNA3.2-A3G.V5. Culture supernatants containing virions were harvested three days post infection and used for infectivity assays on TZM-Bl cells and for immunoblotting.

### Infectivity assay

TZM-bl cells were seeded at a concentration of 1×10^4^ cells per well in 96-well plates and infected with equal volumes of replication-competent virus. Two independent experiments were conducted, and all infections were performed in triplicate. Three days post infection expression of luciferase was analyzed using the Britelite Plus Ultra-High Sensitivity Luminescence Reporter Gene Assay System (PerkinElmer Inc; Waltham, MA) and a Victor X5 Multilabel Plate Reader (PerkinElmer Inc; Waltham, MA). Results were analyzed by one-way ANOVA and Tukey's multiple comparisons test, using PRISM 6.0b (GraphPad Software, Inc., La Jolla, CA.).

### Plasmids

For the APOBEC3G clones, RNA was extracted from rhesus macaque, sooty mangabey, African green monkey and pigtail macaque B-cells and reverse transcribed to synthesize cDNA using the one-step RT-PCR kit (Invitrogen; Carlsbad, CA) and cloned into the pENTR directional TOPO cloning vector (Invitrogen; Carlsbad, CA) according to manufacturer's manual for further sequence analysis. Sequences for independent rhesus macaque alleles (rh1-rh6), sooty mangabey alleles (sm1-sm6) and one pigtail allele (ptA3G) were submitted to Genbank (rh1-rh6: KF020481-KF020486, sm1-sm6: KF020487- KF020492, ptA3G: KF169801). For expression in mammalian cells, the protein coding fragments were transferred into the pcDNA3.2/V5-DEST cloning vector (Invitrogen, Carlsbad, CA) using the Gateway LR Clonase II Enzyme Mix (Invitrogen, Carlsbad, CA).

For Vif-expressing plasmids, *vif* coding sequences were synthesized (GeneArt) and cloned into pcDNA3.1 (Invitrogen, Carlsbad, CA) using PCR to add restriction sites 5′HindIII and 3′NotI as well as adding a C-terminal HA-tag. Vif gene sequences used can be found in GenBank: SIVmac (M33262), SIVsmE041 (HM059825), SIVmac142 (Y00277), SIVmne027 (U79412), SIVmfa186 (KF030930), SIVsmE543 (U72748). The mutant constructs were obtained by site-directed mutagenesis using PfuTurbo Hotstart Polymerase (Agilent Technologies, Santa Clara, CA) and were confirmed by sequence analysis.

Constructs for co-immunoprecipitation (rhA3G^LR^ and rhA3G^Y^) were subcloned into ptr600 plasmids [75]. The A3G constructs were C-terminally labeled with a 3×HA tag by standard overlap PCR. Vif-SIVmac239 and Vif-SIVsm were cloned into the pcrv expression plasmid [76]. The Vif constructs were C-terminally FLAG-tagged by standard overlap PCR. Correct cloned inserts were confirmed by sequencing.

### Sequencing of SIVmfa186 Vif

Retrospective analysis revealed that a pathogenic SIV was freely circulating in crab-eating macaques (*Macaca fascicularis*) housed at the New England Primate Research Center (NEPRC) [41]. An isolate of this virus from animal MF186-76 was obtained from frozen PBMCs, which were co-culture with H9 cells in 1987 [41]. A vial containing cell supernatants from this co-culture was obtained (a gift from R.C. Desrosiers). Viral RNA was isolated using a High Pure Viral RNA Kit (Roche USA, Indianapolis, IN). Specific cDNA products corresponding to the Vif coding sequence were amplified using the SuperScript™ III One-Step RT-PCR System with Platinum® Taq High Fidelity kit (Invitrogen, Carlsbad CA) using following the primers: 647-F AGGGGAGGAATAGGGGATATGACTC and SME041-envR1 R- CACTTAATAGCAAGAGCGCGATAAG. The PCR fragment was then cloned using the TOPO TA cloning kit (Invitrogen: Carlsbad, CA) and sequenced.

### Genotyping

Rhesus macaque and sooty mangabey RNA samples were used for initial screens for polymorphisms in APOBEC3G by RT-PCR of the entire A3G coding sequence, cloning, and sequencing, as described above (see section entitled “Plasmids”). Additional, allele-specific typing was performed by extraction of genomic DNA followed by targeted PCR and direct sequencing. Specifically, APOBEC3G genotypes of rhesus macaques (*Macaca mulatta*) and cynomolgus macaques (*Macaca fascicularis*) (also known as crab-eating macaques) were determined by isolation of genomic DNA from PBMCs using QIAamp DNA Blood Mini Kit (Qiagen; Valencia, CA) and PCR using 100 ng gDNA, Taq polymerase and primers (APOBEC3G-1aF: 5′-GAG GAA AGG AGC TTC AGT GGC AAG A-3′, APOBEC3G-1aR: 5′-GGA GGC CTC AAG AGG GTA AGC AG-3′) with following PCR conditions: 94°C – 1 min, 94°C – 15 sec, 59°C – 30 sec, 68°C – 1 min for a total of 30 cycles, 68°C – 10 min) followed by direct sequencing of a PCR fragment (APOBEC3G-1aF: 5′-GAG GAA AGG AGC TTC AGT GGC AAG A-3′, APOBEC3G-1aR: 5′-GGA GGC CTC AAG AGG GTA AGC AG-3′). PCR fragments were sequenced by Retrogen (San Diego, CA) and data were analyzed with the Codoncode software (Codoncode Corp; Dedham, MA).

### Encapsidation of A3G

Two milliliters of the harvested virus-containing supernatants were used to concentrate virus by ultracentrifugation through 20% sucrose at 35,000 rpm for 75 min at 4°C. The virions were then lysed with 100 µl 2× leammli sample buffer (Sigma, St. Louis, MO) and boiled at 99°C for 10 min. Incorporated A3G and p27 was detected by an immunoblot assay.

### Structure model

A model of the N-terminal active site of residues 1–195 of the rhA3G^LR^ allele was geminated with high confidence using the Protein Homology/analogy Recognition Engine V 2.0 server (PHYRE2) (http://www.sbg.bio.ic.ac.uk/phyre2/html/page.cgi?id=index) [59].

### Accession numbers

Rhesus macaque alleles: rh1-rh6: KF020481- Rh6:KF020486

Sooty mangabey alleles: sm1-sm6: KF020487- KF020492

Pig tailed macaque: KF169801

Vif-SIVmfa186: KF030930

## Supporting Information

Figure S1 **Full alignment of rhA3G and smA3G alleles identified in this study.** The rhesus macaque alleles are indicated with rh1-rh6, the sooty mangabey alleles are indicated with sm1-sm6. The green arrows indicate the polymorphisms in rhesus macaques, the orange arrows indicate polymorphisms in sooty mangabey. The asterisk indicate differences between species.
(TIF)

Figure S2 **Incorporation and antiviral activity of rhesus macaque and sooty mangabey APOBEC3G alleles.** Virions were produced in HEK-293T/17 cells by co-transfection of a full-length SIVmac239ΔVif plasmid and a plasmid coding for one of the six rhesus macaque or the six sooty mangabey alleles or a no A3G control. Infectivity was measured by titration on TZM-bl indicator cells. Error bars indicate the standard deviation of three replicate infections. Immunoblot (bottom) was used to detect incorporation of rhA3G or smA3G in pelleted virions. Virus production was detected via the SIV p27 core antigen using an anti-p27 monoclonal antibody. A3G incorporation into virions was measured by using a V5 specific antibody.
(TIF)

Figure S3 **All sooty mangabey alleles are degraded by Vif-SIVsmE041 and Vif-SIVmac239.** Sooty mangabey A3G allele containing plasmids were co-transfected with a Vif-SIVsmE041 or Vif-SIVmac239 containing plasmid or an empty vector (no Vif) control. The ability of Vif-SIVsmE041 and Vif-SIVmac239 to induce A3G degradation is shown. Anti-β-actin served as a protein loading control.
(TIF)

Figure S4 **A polymorphism in rhesus macaques at position 130 influences the ability of the rhA3G^LR^ allele to resist Vif-SIVsmE041 induced degradation.** (A) Site directed mutagenesis was used to introduce a N130D mutation into either one of the three rhA3G alleles (rhA3G^LR^, rhA3G^LL^ or rhA3G^Y^). Their ability to resist Vif-mediated degradation was visualized by western blot. We used an empty vector control to test expression in the absence of Vif (indicated as “control”). Anti-β-actin served as a protein loading control. (B) Genotyping frequencies of 130D/N (n = 219). (C) Frequencies of 130D and 130N among LR homozygotes.
(TIF)

Figure S5 **Partial alignment of the NTD of Vif.** Depicted is an alignment of the first 56 amino acids of the viral Vif protein from SIVs from different species. The sequences labeled with accession numbers, including SIVsmCFU212, represent Vifs from independently isolated SIVsm strains [42]. Highlighted in red is residue 17, which is a negatively charged glutamic acid in most macaque derived SIV strain Vifs and an uncharged glycine in Vif proteins derived from HIV-2, SIVstm, SIVsmE041 and several other SIVsm strains.
(TIF)

Figure S6 **Other SIVsm isolates behave like Vif-SIVsmE041.** Immunoblot showing activity of Vif-SIVsmPBj, Vif-SIVsm-PG, Vif-SIVsmCFU212 or an empty vector (no Vif) control against the three rhA3G alleles (rhA3G^LR^, rhA3G^LL^ or rhA3G^Y^). Anti-β-actin served as a protein loading control.
(TIF)
