# Supplementary figures and images for: APOBEC3G Polymorphism as a Selective Barrier to Cross-Species Transmission and Emergence of Pathogenic SIV and AIDS in a Primate Host
Source: PLoS Pathog. 2013 Oct 3;9(10):e1003641. doi: 10.1371/journal.ppat.1003641 (PMC3789815; doi:10.1371/journal.ppat.1003641)

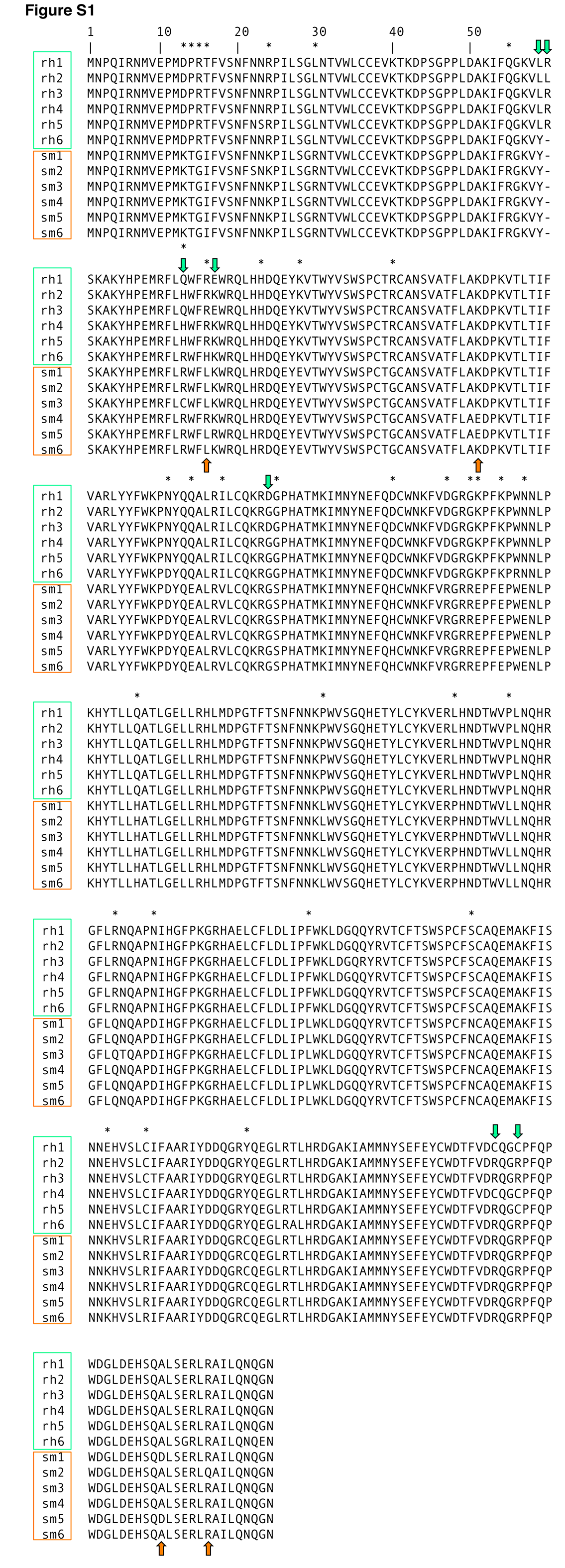

Supplement: Figure S1 — Full alignment of rhA3G and smA3G alleles identified in this study. The rhesus macaque alleles are indicated with rh1-rh6, the sooty mangabey alleles are indicated with sm1-sm6. The green arrows indicate the polymorphisms in rhesus macaques, the orange arrows indicate polymorphisms in sooty mangabey. The asterisk indicate differences between species. (TIF) [file ppat.1003641.s001.tif]

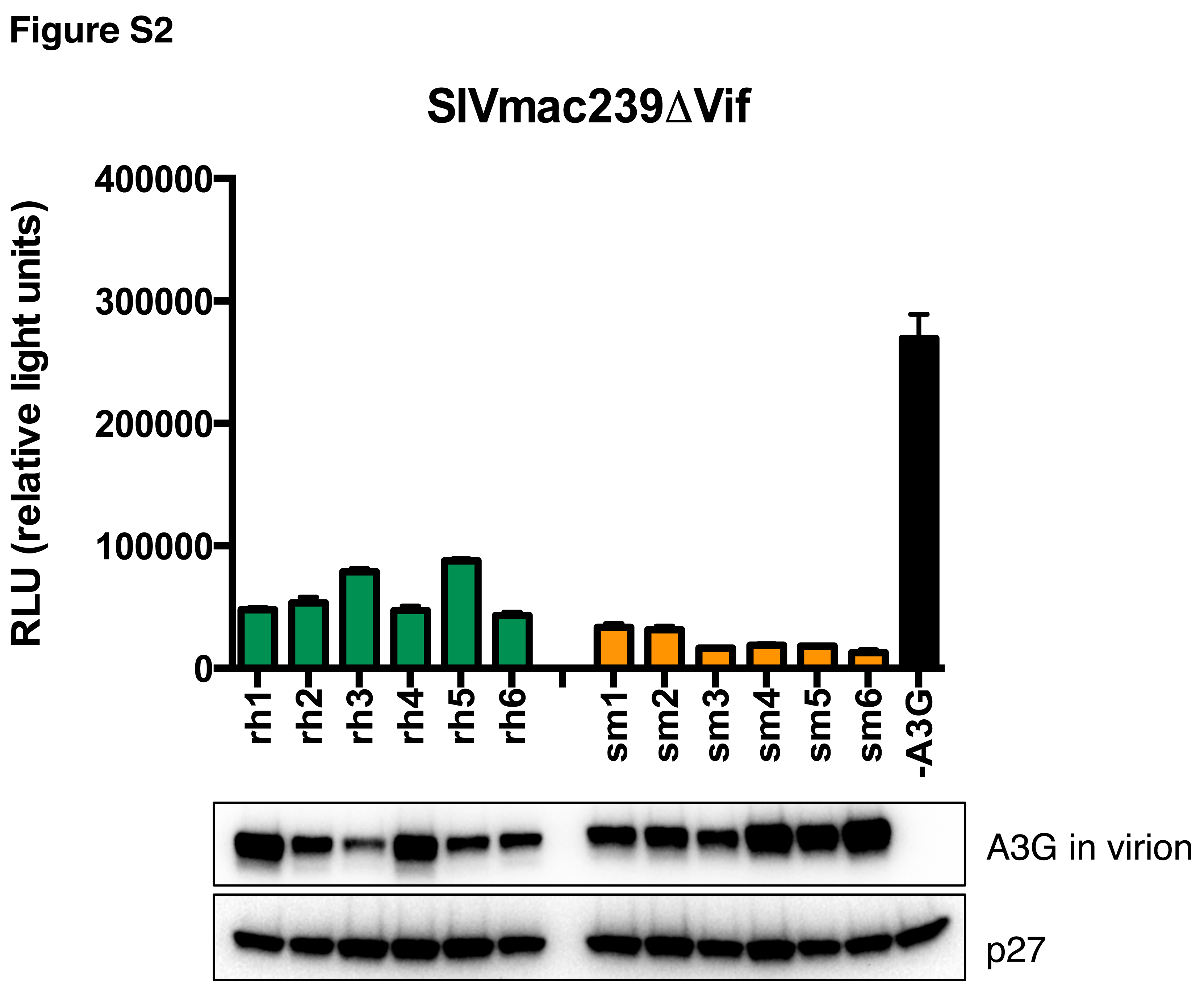

Supplement: Figure S2 — Incorporation and antiviral activity of rhesus macaque and sooty mangabey APOBEC3G alleles. Virions were produced in HEK-293T/17 cells by co-transfection of a full-length SIVmac239ΔVif plasmid and a plasmid coding for one of the six rhesus macaque or the six sooty mangabey alleles or a no A3G control. Infectivity was measured by titration on TZM-bl indicator cells. Error bars indicate the standard deviation of three replicate infections. Immunoblot (bottom) was used to detect incorporation of rhA3G or smA3G in pelleted virions. Virus production was detected via the SIV p27 core antigen using an anti-p27 monoclonal antibody. A3G incorporation into virions was measured by using a V5 specific antibody. (TIF) [file ppat.1003641.s002.tif]

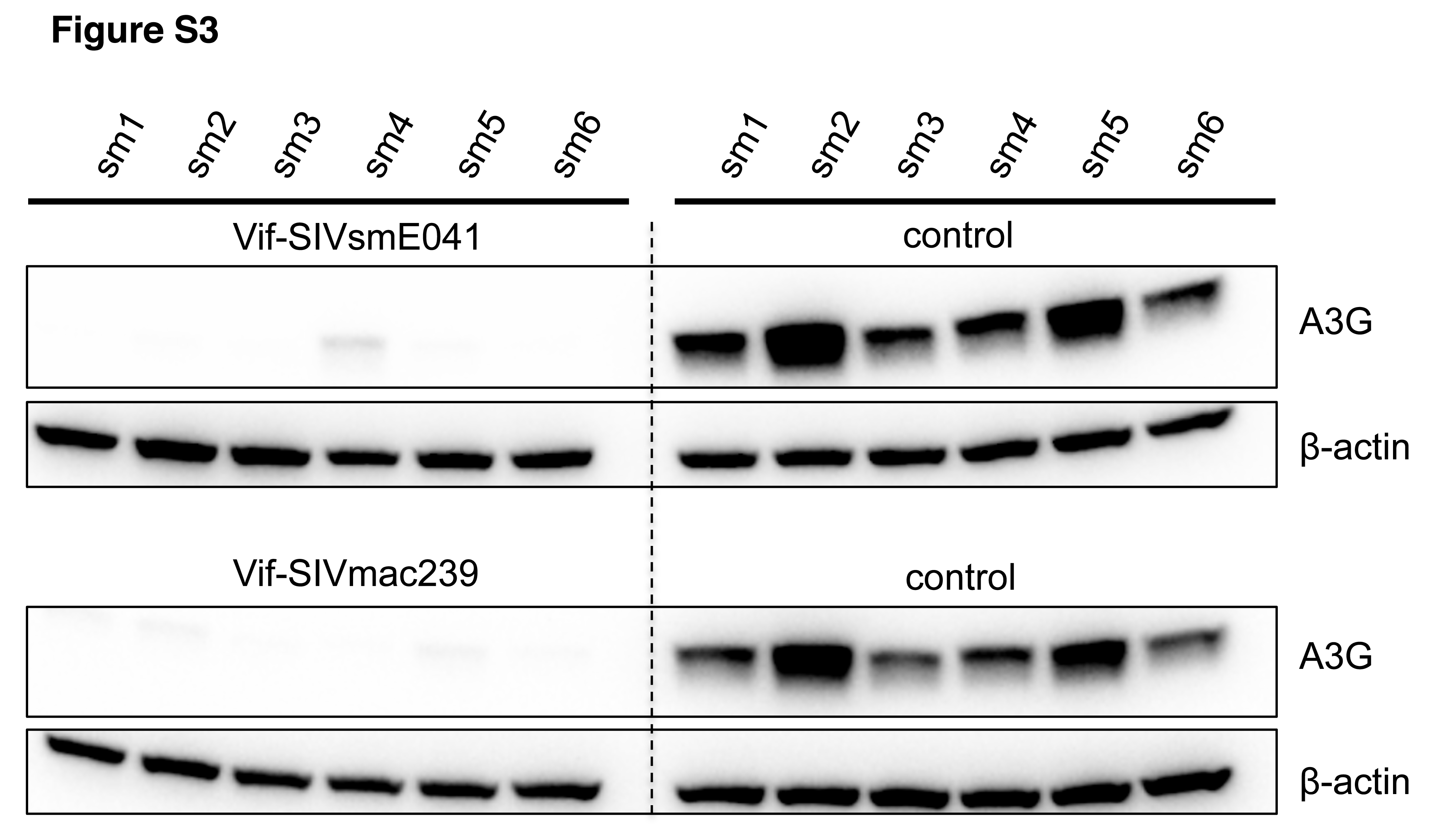

Supplement: Figure S3 — All sooty mangabey alleles are degraded by Vif-SIVsmE041 and Vif-SIVmac239. Sooty mangabey A3G allele containing plasmids were co-transfected with a Vif-SIVsmE041 or Vif-SIVmac239 containing plasmid or an empty vector (no Vif) control. The ability of Vif-SIVsmE041 and Vif-SIVmac239 to induce A3G degradation is shown. Anti-β-actin served as a protein loading control. (TIF) [file ppat.1003641.s003.tif]

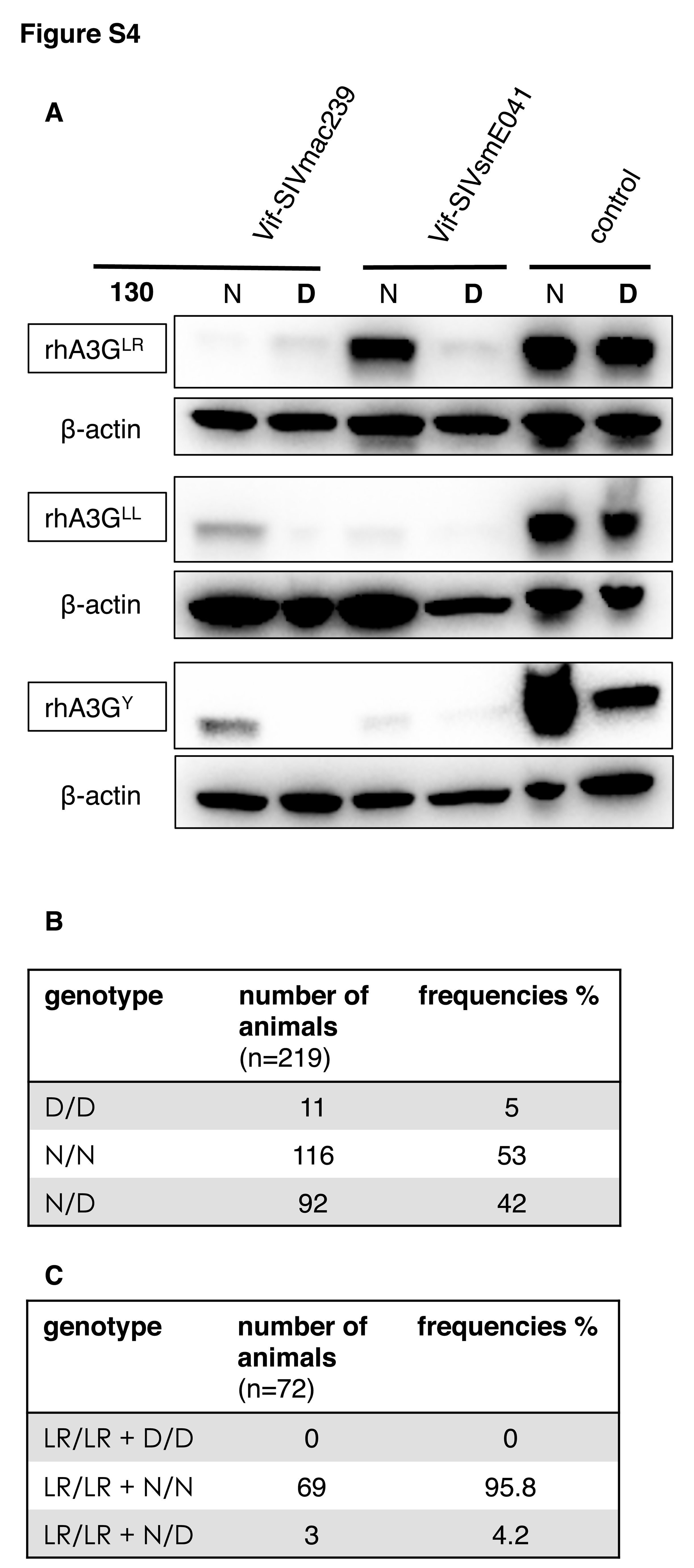

Supplement: Figure S4 — A polymorphism in rhesus macaques at position 130 influences the ability of the rhA3GLR allele to resist Vif-SIVsmE041 induced degradation. (A) Site directed mutagenesis was used to introduce a N130D mutation into either one of the three rhA3G alleles (rhA3GLR, rhA3GLL or rhA3GY). Their ability to resist Vif-mediated degradation was visualized by western blot. We used an empty vector control to test expression in the absence of Vif (indicated as “control”). Anti-β-actin served as a protein loading control. (B) Genotyping frequencies of 130D/N (n = 219). (C) Frequencies of 130D and 130N among LR homozygotes. (TIF) [file ppat.1003641.s004.tif]

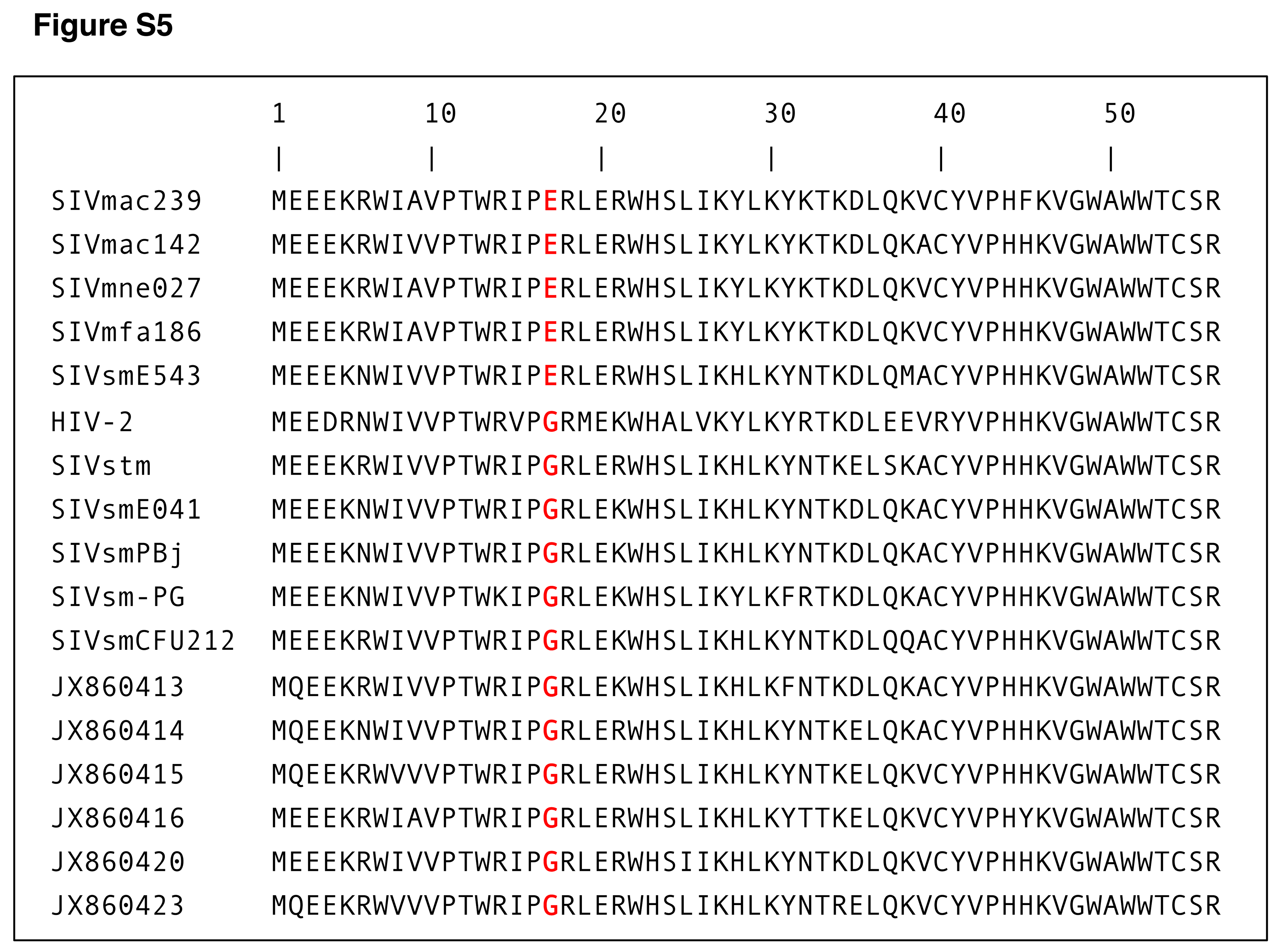

Supplement: Figure S5 — Partial alignment of the NTD of Vif. Depicted is an alignment of the first 56 amino acids of the viral Vif protein from SIVs from different species. The sequences labeled with accession numbers, including SIVsmCFU212, represent Vifs from independently isolated SIVsm strains [42]. Highlighted in red is residue 17, which is a negatively charged glutamic acid in most macaque derived SIV strain Vifs and an uncharged glycine in Vif proteins derived from HIV-2, SIVstm, SIVsmE041 and several other SIVsm strains. (TIF) [file ppat.1003641.s005.tif]

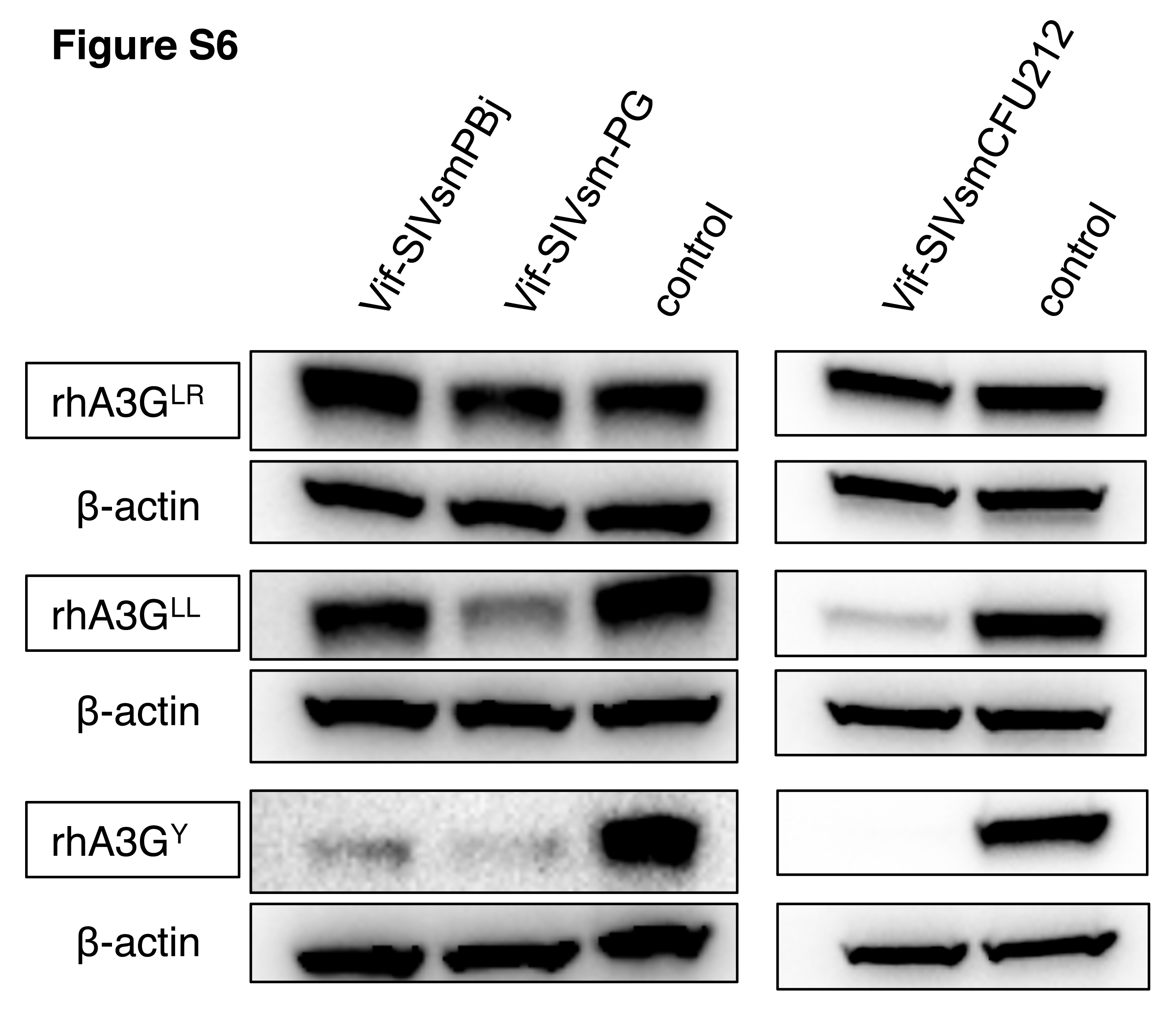

Supplement: Figure S6 — Other SIVsm isolates behave like Vif-SIVsmE041. Immunoblot showing activity of Vif-SIVsmPBj, Vif-SIVsm-PG, Vif-SIVsmCFU212 or an empty vector (no Vif) control against the three rhA3G alleles (rhA3GLR, rhA3GLL or rhA3GY). Anti-β-actin served as a protein loading control. (TIF) [file ppat.1003641.s006.tif]
